# Supplementary material for: Relation between Dietary Habits, Physical Activity, and Anthropometric and Vascular Parameters in Children Attending the Primary School in the Verona South District
Source: Nutrients. 2019 May 14;11(5):1070. doi: 10.3390/nu11051070 (PMC6566536; doi:10.3390/nu11051070)
Supplement: Supplementary file 1 [file nutrients-11-01070-s001.pdf]

1. Supplementary methods

1.1. Computation of MET minutes/week

First item of PAQ-C was itself composed by questions about 21 moderate-intensive activities and minutes spent on them.
From Compendium of Physical Activity (https://sites.google.com/site/compendiumofphysicalactivities/home) [1] a MET value for each activity has been assigned and multiplied for the time (min) children took part on them, obtaining a MET-minutes/week value. [2].

1.2. Extrapolation of dietary data from FFQ

Dietary data have been converted into energy intake (kcal/die) using as references two Italian food composition tables: the one proposed by *"Consiglio per la ricerca in agricoltura e l'analisi dell'economia agraria, CREA" (http://nut.entecra.it/646/tabelle_di_composizione_degli_alimenti.html) and the one provided by "Food Composition Database for Epidemiological Studies in Italy" by Gagnarella P, Salvini S Parpinel M. (Version 1.2015 Website http://www.bda-ieo.it/*).
Since FFQ didn't provide information about portion size we used standard size proposed by Italian Society of Human Nutrition [(*SINU), S.I.d.N.U. Iv Revisione dei Livelli di Assunzione di Riferimento di Nutrienti ed Energia per la Popolazione Italiana (Larn). Available online: http://www.sinu.it/html/pag/tabelle_larn_2014_rev.asp*] [3].
Food usually consumed as meal have been used: cereals and tubers, dairy products, legumes, fish, vegetables, fresh and dried fruit, meat, FFQ category of fast food, sweets, eggs. PCA is a multivariate technique used to represent a set of inter-correlated quantitative dependent variables into a set of new orthogonal variables called "principal components" [4]. In this case PCs are represented by pattern of food groups consumption that explain the maximum amount of variance [5]. The result of PCA is a factor loading matrix from which the components are derived and a factor score for each individual derived by summing the individual intakes of the food item

weighed by standardized coefficient for each pattern. These scores, one for each pattern, are used in either correlation or regression analysis to examine relationships between various eating patterns and the outcome of interest [6]. Principal Component Analysis (PCA) has been performed using a correlation matrix with varimax rotation. Only eigenvalues greater than 1 has been retained, and only loading scores >|200| has been considered contributing to the pattern.

2. Supplementary tables

Suppl. Table 1. Difference in food intake and physical activity between overweight (BMI> 85° percentile for age) or obese (BMI > 95° percentile for age) and normal weight.

	**Overweight or Obese n=103**	**Normal Weight n=197**	
	mean±SD	*mean±SD*	**p-value**
**Fast Food**	1.5±0.5	1.4±0.6	n.s.
**Cereals and Tubers**	1.9±0.5	2.0±0.4	n.s.
**Vegetables**	2.1± 1.0	2.3± .9	**p<0.05**
**Fruit**	1.6± .9	1.5±0.9	n.s.
**Eggs**	1.9±1.0	1.9±0.9	n.s.
**Meat**	1.7±0.6	1.6±0.6	n.s.
**Dairy product**	1.9±0.6	1.9±0.7	n.s.
**Sweets**	1.9±0.7	2.0±0.7	n.s.
**Legumes**	1.3±1.1	1.4±1.1	n.s.
**Fish**	0.9±0.5	1.0±.5	n.s.
**Nuts**	0.8±1.1	0.9±1.1	n.s.
**Animal-derived fat**	0.7±0.6	0.7±0.7	n.s.
**Evo oil**	4.0±1.1	3.8±1.4	n.s.
**Seed oil**	1.5±1.4	1.2±1.4	n.s.
**Daily energy intake (kcal/die)**	2886.14±1040.2	3076.6±1243.5	n.s.
**PAQ-C score**	1.8±0.5	1.9±0.5	n.s.
**Moderate-vigorous activity (MET-min/wk)**	2892.6±4084.2	3582.0±3894.8	n.s.

Suppl. Table 2. Difference in food intake and physical activity between normal-high BP (Brachial SBP or Brachial DBP>90° percentile) or high BP (Brachial SBP or Brachial DBP>95°) and normal BP group.

	**Normal-high BP or high BP n=118**	**Normal BP n=182**	
	mean±SD	*mean±SD*	*p-value*
**Fast Food**	1.4±0.6	1.4±0.5	n.s.
**Cereals and Tubers**	2.0±0.5	2.0±0.4	n.s.
**Vegetables**	2.1±1.0	2.3±0.9	**p<0.05**
**Fruit**	1.5±0.9	1.5±0.9	n.s.
**Eggs**	1.9±0.8	0.9±1.0	n.s.
**Meat**	1.7±0.6	1.6±0.5	n.s.
**Dairy product**	1.9±0.7	1.9±0.7	n.s.
**Sweets**	2.0±0.7	1.9±0.7	n.s.
**Legumes**	1.3±1.1	1.5±1.0	n.s.
**Fish**	1.0±0.6	1.0±0.5	n.s.
**Nuts**	0.8±1.0	0.9±1.1	n.s.
**Animal-derived fat**	0.7±0.6	0.8±0.7	n.s.
**Evo oil**	3.8±1.2	3.9±1.3	n.s.
**Seed oil**	1.4±1.5	1.3±1.4	n.s.
**Daily energy intake (kcal/die)**	3079.0±1412.8	2967.3±1001.3	n.s.
**PAQ-C score**	1.8±0.5	1.8±0.5	n.s.
**Moderate-vigorous activity (MET-min/wk)**	3689.2± 4943.9	3146.0± 3245.1	n.s.

Suppl. Table 3. Factor loadings >|200| associated to the dietary pattern.

	**1st Pattern**	**2nd Pattern**
**Eigenvalue**	3.25	1.39
**Variance explained**	32.5%	13%
Fish	**0.577**	0.402
Legumes	**0.627**	-
Vegetables	**0.781**	-
Fresh and dried fruit	**0.715**	-
Dairy Products	**0.486**	0.281
Cereals and tubers	0.403	**0.410**
Sweets	-	**0.705**
Fast Food	-	**0.731**
Meat	0.204	**0.737**
Eggs	0.274	**0.407**

Factor loadings of the food groups in the two patterns derived from PCA. Only factor>|0.200| has been retained. Highest factor scores for each variable are expressed in bold.

Suppl. Table 4. Correlation between dietary pattern and anthropometric, hemodynamic and gluco-lipid parameters.

	**"Healthy" pattern**	**"Unhealthy" pattern**
**BMI kg/m²**	-0.049	0.059
**Z-score BMI**	-0.067	0.059
**Waist-height ratio**	-0.086	-0.093
**Z-score waist-height ratio**	0.031	0.032
**Brachial SBP mmHg**	0.064	0.027
**Z-score Brachial SBP**	0.051	0.008
**Brachial DBP mmHg**	-0.044	**0.130***
**Z-score Brachial DBP**	-0.069	**0.130***
**Central SBP mmHg**	-0.057	0.096
**Z-score Central SBP**	0.050	0.036
**PWV m/s**	0.006	0.113
**Z-score PWV**	-0.070	0.097
**Capillary Cholesterol mg/dl**	-0.056	0.001
**Capillary Triglycerides mg/dl**	-0.121	-0.002
**Capillary Glucose mg/dl**	**-0.191****	-0.013

Significant Spearman correlations are expressed in bold (*= p-value<0.05; **=p-value<0.01)

Suppl. Table 5. Correlation between physical activity, expressed in PAQC score and MET-min/week, and anthropometric, hemodynamic and gluco-lipid parameters.

	**PAQC Score**	**MET (min/wk)**
**BMI kg/m^2**	-0.016	-0.086
**Percentile BMI-age**	-0.019	-0.091
**Waist-height ratio**	-0.040	-0.095
**Z-score Waist-height ratio**	-0.036	-0.114
**PWV m/s**	-0.045	-0.056
**Brachial SBP mmHg**	0.063	-0.012
**Percentile Brachial SBP**	0.031	-0.026
**Brachial DBP mmHg**	-0.012	**-0.142***
**Percentile Brachial DBP**	-0.019	**-0.141***
**Central SBP mmHg**	-0.004	**-0.141***
**Z-Score Central SBP-height**	-0.029	**-0.171****
**Capillary Cholesterol mg/dl**	0.043	0.046
**Capillary Triglycerides mg/dl**	-0.049	**-0.157***
**Capillary Glucose mg/dl**	0.071	-0.026

Significant Spearman correlations are expressed in bold (*= p-value<0.05; **=p-value<0.01)

Suppl. Table 6. Difference of anthropometric, hemodyniamic and gluco-lipidic parameters among categories of "low", "medium" and "high" adherence to moderate-vigorous physical activity.

	"Low" n=44	"Medium" n=143	"High" n=101	
	mean±SD	*mean±SD*	*mean±SD*	***p-value***
**BMI kg/m²**	18.6±3.3	18.1±3.8	17.9±3.0	n.s.
**Waist-height ratio**	0.46±0.09	0.45±7 0.09	0.454±0.05	n.s.
**Brachial SBP mmHg**	110.4±9.9	109.7±10.2	110.1±9.3	n.s.
**Brachial DBP mmHg**	69.1±7.9	66.6±7.9	65.8±7.5	n.s.
**PWV m/s**	4.6±0.6	4.6±0.8	4.5±11	n.s.
**Central SBP mmHg**	104.9±10.1*°	100.7±9.5	99.9±9.5	**0.016**
**Capillary Cholesterol mg/dl**	190.5±63.2	172.0±79.0	167.5±68.7	n.s.
**Capillary Triglycerides mg/dl**	231.8±34.6	229.7±39.9	235.3±39.0	n.s.
**Capillary Glucose mg/dl**	88.6±9.1	88.9±9.8	88.0±11.0	n.s.

Legends: Categories: "*Low*": <600 MET-min/wk; "*Medium*": 600-3000 MET-min/wk; "*High*": >3000 MET-min/wk; *: "*Low*" vs "*Medium*"; °: "Low" vs "High"; §: "*Medium*" vs "*High*".

Suppl. Table 7 (a-f). Multivariate models including dietary intake and physical activity in relation to anthropometric, hemodynamic and gluco-lipidic parameters.

Dependent variable: **PWV m/s**					
	β	SE	p value	CI 95%	
**Age yrs**	0.088	0.073	n.s.	-0.054	0.231
**Sex**	-0.048	0.106	n.s.	-0.256	0.160
**Ethnicity**	0.056	0.118	n.s.	-0.177	0.290
**BMI, kg/m²**	0.014	0.015	n.s.	-0.016	0.045
**Daily energy intake (quartiles)**	-0.053	0.053	n.s.	-0.158	0.051
**PAQ-C Score (quartiles)**	0.027	0.047	n.s.	-0.066	0.120
**Fast Food intake**	0.337	0.100	**p<0.01**	0.140	0.534

Suppl. Table 7a

Dependent variable: **Glucose mg/dL**					
	β	SE	p value	CI 95%	
**Age yrs**	1.401	0.912	n.s.	-0.397	3.200
**Sex**	4.593	1.286	**p<0.001**	2.058	7.127
**Ethnicity**	-4.986	1.447	**p<0.01**	-7.838	-2.133
**BMI, kg/m²**	0.298	0.182	n.s.	-0.061	0.657
**Daily energy intake (quartiles)**	0.231	0.659	n.s.	-1.067	1.529
**PAQ-C Score (quartiles)**	0.484	0.587	n.s.	-0.673	1.641
**Vegetables intake**	-1.729	0.772	**p<0.05**	-3.251	-0.207

Suppl. Table 7 b

Dependent variable: **Glucose mg/dL**					
	β	SE	p value	CI 95%	
**Age yrs**	1.709	0.919	n.s.	-0.103	3.521
**Sex**	4.410	1.281	**p<0.01**	1.886	6.934
**Ethnicity**	-3.729	1.463	**p<0.05**	-6.611	-.846
**BMI, kg/m²**	0.401	0.183	**p<0.05**	0.039	0.762
**Daily energy intake (quartiles)**	0.313	0.645	n.s.	-0.959	1.585
**PAQ-C Score (quartiles)**	0.593	0.585	n.s.	-0.559	1.746
**Fruit intake**	-2.530	0.885	**p<0.05**	-4.274	-0.785

Suppl. Table 7 c

Dependent variable: **Glucose mg/dL**					
	**β**	**SE**	**p value**	**CI 95%**	
**Age yrs**	1.729	0.937	n.s.	-0.118	3.575
**Sex**	4.353	1.293	**p<0.01**	1.804	6.901
**Ethnicity**	-4.031	1.459	**p<0.01**	-6.908	-1.154
**BMI, kg/m²**	0.356	0.183	n.s.	-0.005	0.718
**Daily energy intake (quartiles)**	-0.078	0.619	n.s.	-1.298	1.142
**PAQ-C Score (quartiles)**	0.445	0.594	n.s.	-0.725	1.616
**Nuts intake**	-1.255	0.631	**p<0.05**	-2.498	-0.011

Suppl. Table 7 d

Dependent variable: **Cholesterol mg/dL**					
	**β**	**SE**	**p value**	**CI 95%**	
**Age yrs**	-8.800	4.019	**p<0.05**	-16.734	-0.865
**Sex**	17.267	5.807	**p<0.01**	5.800	28.733
**Ethnicity**	-11.442	6.355	n.s.	-23.989	1.105
**BMI, kg/m²**	-0.686	0.842	n.s.	-2.348	0.975
**Daily energy intake (quartiles)**	-4.375	2.831	n.s.	-9.963	1.214
**PAQ-C Score (quartiles)**	-0.093	2.581	n.s.	-5.189	5.004
**Animal fat intake**	11.009	4.893	**p<0.05**	1.349	20.669

Suppl. Table 7 e

Dependent variable: **Glucose mg/dl**					
	**β**	**SE**	**p value**	**CI 95%**	
**Age yrs**	0.233	0.075	0.002	0.085	0.380
**Sex**	- 0.129	0.109	0.238	- 0.344	0.086
**Ethnicity**	- 0.157	0.121	0.195	- 0.396	0.081
**BMI, kg/m²**	0.004	0.015	0.810	- 0.026	0.033
**Daily energy intake (quartiles)**	0.459	0.050	0.000	0.360	0.558
**PaQ-C Score (quartiles)**	0.064	0.048	0.190	- 0.032	0.159
**"Healthy" pattern**	- 0.016	0.006	**p<0.01**	- 0.027	- 0.005

Suppl. Table 7 f

Dependent variable: **Brachial DBP mg/dl**					
	**β**	**SE**	**p value**	**CI 95%**	
**Age yrs**	-1.429	0.629	**p<0.05**	-2.668	-0.190
**Sex**	-0.853	0.917	0.353	-2.658	0.952
**Ethnicity**	0.440	0.130	**p<0.01**	0.184	0.695
**BMI, kg/m²**	1.135	1.020	0.267	-0.873	3.144
**Daily energy intake (quartiles)**	0.232	0.512	0.651	-0.776	1.241
**PaQ-C Score (quartiles)**	0.237	.0411	0.565	-0.572	1.045
**"Unhealthy" pattern**	0.911	0.539	0.092	-0.150	1.972

Suppl. Table 7 g

3. Supplemental bibliography

1. Ainsworth, B. E.; Haskell, W. L.; Herrmann, S. D.; Meckes, N.; Bassett, D. R.; Tudor-Locke, C.; Greer, J. L.; Vezina, J.; Whitt-Glover, M. C.; Leon, A. S. 2011 Compendium of Physical Activities. *Med. Sci. Sport. Exerc.* **2011**, *43*, 1575–1581, doi:10.1249/MSS.0b013e31821ece12.

2. IPAQ Research Committee. Guidelines for data processing and analysis of the international physical Activity questionnaire (IPAQ)—short and long form scoring Available online: https://sites.google.com/site/theipaq/ (accessed on Nov 14, 2018).

3. *Livelli di Assunzione di Riferimento di Nutrienti ed energia per la popolazione italiana IV Revisione*;

4. Herv´, H.; Abdi, H.; Williams, L. J. Principal component analysis. **2010**, doi:10.1002/wics.101.

5. Tucker, K. L. Dietary patterns, approaches, and multicultural perspectiveThis is one of a selection of papers published in the CSCN–CSNS 2009 Conference, entitled Can we identify culture-specific healthful dietary patterns among diverse populations undergoing nutrition. *Appl. Physiol. Nutr. Metab.* **2010**, *35*, 211–218, doi:10.1139/H10-010.

6. Hu, F. B. *Dietary pattern analysis: a new direction in nutritional epidemiology*;
